# Editorial: Innovative behavior in entrepreneurship: Analyzing new perspectives and challenges

**DOI:** 10.3389/fpsyg.2023.1123236

**Published:** 2023-01-19

**Authors:** Jose Ramon Saura, Daniel Palacios-Marqués, Marisol B. Correia, Belem Barbosa

**Affiliations:** ^1^Department of Business Economics, Rey Juan Carlos University, Madrid, Spain; ^2^Business Organization Department, Universitat Politècnica de València, Valencia, Spain; ^3^ESGHT, Universidade do Algarve, Faro, Portugal; ^4^Centre for Tourism Research, Development, and Innovation – CiTUR, Faro, Portugal; ^5^Research Centre for Tourism, Sustainability and Well-Being – CinTurs, Faro, Portugal; ^6^CEG-IST, Instituto Superior Técnico, Universidade de Lisboa, Lisbon, Portugal; ^7^School of Economics and Management, University of Porto, Porto, Portugal

**Keywords:** innovative behavior, entrepreneurship, innovation, online behavior, innovation and research

In recent years, the relationship between behavior and innovation has come to be globally accepted as a prerequisite of business success (Li et al., 2022). Innovative behavior is seen as an introduction to the application and development of new ideas, processes, initiatives, or actions by qualified professionals (RoŽman and Štrukelj, 2021). Developed either individually or collectively, innovative behavior drives creativity and is directly linked to a multitude of behaviors that lead to the generation of new ideas, initiatives, and value for new companies' products and services (Barbosa et al., 2022).

In this sense, it is important to highlight the importance of entrepreneurial projects in the development of the economy or new business models. Innovation and online behavior are essential variables to promote the creation of new forms of business and behavior analysis in digital ecosystems (Ribeiro-Navarrete et al., 2021; Saura et al., 2022). This Research Topic has identified a gap in the literature concerning how human beings process and acquire new skills for work relationships that promote innovative behavior, and particularly if its development is focused on new business models, entrepreneurship projects, or innovative startups models (Saura et al., 2021a). To date, there have emerged different theories on how business management should promote innovative behavior to increase the value of their products and services (Yang, 2022). However, available literature on the forms of managing innovative behavior in new and entrepreneurship projects remains scarce (LiŽbetinová et al., 2022).

In this way, this Research Topic has been focused on understanding new forms of behavior characteristics, motivations, perceived skills in changing contexts, organization and leadership at work, role of innovation in the behavior of individuals and work groups. Similarly, it is necessary to analyze the creation of critical knowledge linked to a global economy and to better understand the role of innovation and behavior in companies' success.

In this way, authors such as Zhang, Liu et al. reveal the link between social information processing theory in the orientation of CEOs to entrepreneurs. They point out that the internal reasons for the interpretation of information by middle management teams (MMTs) is critical for the correct coordination of the behaviors of entrepreneurs linked to innovation. In another contribution to this special issue, Wu W. et al. focus their attention on the analysis of perceived environmental corporate social responsibility (ECSR) and employee's innovative behavior. In their study, they identify positive results in relation to the perceived ECSR and organizational identification. In addition, this result encourages the innovative behavior of employees to positively influence the business organization. In addition, they discuss the influence of innovative behavior levels on the trust of employees in a company.

Also, Tang S. et al. analyze innovations in small and medium-sized enterprises (SMEs) from a dynamic capabilities' perspective. In their study, they conclude and define the links between complex causal relationship and environmental turbulence, absorptive capacity, and SMEs' radical innovation. The implications help to understand the processes of innovative behavior in SMEs. Likewise, Wu M. et al. focus their attention on understanding the role of open innovation on the adoption of innovative behaviors in companies. They show the influence of mergers and acquisitions (M&A) as one of the main ways for enterprises to obtain knowledge and technology.

In the contribution presented by Yuan and Liu the role of perceived support for innovation lead to deviant innovation behavior of knowledge workers is analyzed. The findings suggest that perceived support for innovation can significantly predict deviant innovation behavior; innovation commitment fully mediates the relationship between perceived support for innovation and deviant innovation behavior; public threat to self-identity plays a moderating role in the relationship between innovation commitment and deviant innovation behavior; and public threat to self-identity moderates the mediating effect of innovation commitment on perceived support for innovation and deviant innovation behavior.

Likewise, in the study by Tang Y. et al. innovation performance is studied from a competition perspective. The authors identify the relationship between employees' strong growth need and leader–members. The main conclusion is that this relationship gets weaker for supervisors with higher perceived status threat. In addition, this relationship drives innovation performance due to its link with competition and leadership status. In the same line of research, Chen and Liu reveal the organizational effects linked to the commitment for innovative behavior in companies. They focus on understanding the existing relationships between the team-member exchange as an effective measure to boost innovation. They conclude the importance of promoting these work methodologies at many levels of the organization.

In this context, Wang et al. study the link between innovative behavior of employees and the support of the organization's leaders and their wellbeing at work. Thus, based on social comparison theory and social exchange theory, the study results demonstrate that employee innovative behavior is directly and positively related to workplace wellbeing, employee innovative behavior is indirectly and positively related to workplace wellbeing through leader support for innovation, and finally, the negative association between employee innovative behavior and workplace wellbeing *via* coworker ostracism is unsupported.

Also focusing on innovation and leadership, Liu et al. develop a study in which they highlight the importance of the impact of self-serving leadership on employee innovation behavior. They study the roles of workplace anxiety and team psychological safety concluding that self-serving leadership is negatively correlated with employee innovation behavior, and the team psychological safety and workplace anxiety mediated this relationship. Likewise, García de Blanes Sebastián et al. reveal a model that uses UTAUT2 to determine behavioral intention factors in the use of the artificial intelligence (AI) virtual assistants in organizations (Saura et al., 2021b). The main study results reveal that factors, such as habit, trust, and personal innovation, have a significant impact on the adoption of virtual assistants. However, on the other side, performance expectancy, effort expectancy, facilitating conditions, social influence, hedonic motivation, price/value, and perceived privacy risk were not significant factors in the users' intention to adopt this service. These results are important since the adoption of virtual assistants is directly linked to innovative behavior in companies.

Another contribution to this collection is that of Hao et al. who study if venture capital cross-border syndication spur corporate innovation. The results of their study deepen the understanding of the relationship between venture capital (VC) cross-border syndication and corporate innovation and provide essential guidance to domestic VC firms promoting corporate innovation in open partnerships. Likewise, Hu et al. focus their attention on understanding how the capability reconfiguration impacts the innovation performance. Their study contributes to the theory of dynamic capability and catch-up by revealing how innovation magnitude affects capability reconfiguration and subsequent innovation performance in different catch-up stages.

Likewise, de Jaureguizar Cervera et al. focus on the economic factors of innovation and behavior by studying the factors affecting short-term rental first price. They link the results considering some of the factors affecting the psychological behavior of tourism consumers. Focused on the rural tourism area, Zhang, Sun et al. they present a study to understand the factors of poverty reduction in rural areas as an important development goal concerned by the international community. As a contribution, they propose a conceptual framework for the sustainable development of social entrepreneurship and enriches the research on the process of realizing social opportunities in social entrepreneurship. Finally, Jia et al. also analyze the relationship between executive poverty experience and innovation performance. They conclude that the impact of executive poverty experience on innovation performance is more significant in fierce market competition and provide empirical evidence for improving corporate innovation performance.

Finally, this Research Topic offers insights to specifically understand innovative behavior in entrepreneurship. The contributions published in this Research Topic identify gaps and propose future lines of research to solve future the challenges and boost new opportunities in this research field.

## Author contributions

JS drafted this contribution. DP-M, MC, and BB revised and modified this contribution. All authors contributed to the article and approved the submitted version
